# High prevalence of cervical high-risk human papillomavirus infection mostly covered by Gardasil-9 prophylactic vaccine in adult women living in N’Djamena, Chad

**DOI:** 10.1371/journal.pone.0217486

**Published:** 2019-06-03

**Authors:** Ralph-Sydney Mboumba Bouassa, Zita Aleyo Nodjikouambaye, Damtheou Sadjoli, Chatté Adawaye, Hélène Péré, David Veyer, Mathieu Matta, Leman Robin, Serge Tonen-Wolyec, Ali Mahamat Moussa, Donato Koyalta, Laurent Belec

**Affiliations:** 1 Ecole Doctorale Régionale d’Infectiologie Tropicale de Franceville, Franceville, Gabon; 2 Laboratoire de Virologie, Hôpital Européen Georges Pompidou, Faculté de Médecine Paris Descartes, Université Paris Descartes (Paris V), Sorbonne Paris Cité, Paris, France; 3 Service de Gynécologie-Obstétrique, Hôpital de la Mère et de l’Enfant, N’Djamena, Chad; 4 Cabinet Médical de Gynécologie Obstétrique “La Renaissance Plus,” N’Djamena, Chad; 5 Faculté des Sciences de la Santé Humaine, Université de N’Djamena, N’Djamena, Chad; 6 Institut National Supérieur des Sciences et Techniques d’Abéché, Abéché, Chad; 7 Faculté de Médecine, Université de Bunia, Bunia, Democratic Republic of the Congo; 8 Faculté de Médecine et de Pharmacie, Université de Kisangani, Kisangani, Democratic Republic of the Congo; 9 Service de Gastro-entérologie, Hôpital Général de Référence Nationale, N’Djamena, Chad; 10 UNAIDS, N’Djamena, Chad; IAVI, UNITED STATES

## Abstract

**Background:**

We conducted in 2018 a descriptive, quantitative, population-based, cross-sectional survey estimating the prevalence of cervical high-risk human papillomavirus (HR-HPV) infection and associated risk factors among adult women living in N’Djamena, Chad.

**Methods:**

Five of the 10 districts of N’Djamena were randomly selected for inclusion. Peer educators contacted adult women in community-churches or women association networks to participate in the survey and come to the clinic for women’s sexual health “*La Renaissance Plus*”, N’Djamena. Medical, socio-demographical and behavioral informations were collected. HPV DNA was detected and genotyped in endocervical swab using Anyplex II HPV28 genotyping test (Seegene, Seoul, South Korea).

**Results:**

253 women (mean age, 35.0 years; range, 25–65) including 3.5% of HIV-positive women were prospectively enrolled. The prevalence of HPV infection was 22.9%, including 68.9% of HR-HPV infection and 27.6% being infected with multiple genotypes, providing a total HR-HPV prevalence of 15.8% (95% CI%: 11.3–20.3). The most prevalent HR-HPV genotypes were HPV-58, HPV-35, HPV-56, HPV-31, HPV-16, HPV-45, HPV-52 and HPV-18. HPV types targeted by the prophylactic Gardasil-9 vaccine were detected in nearly 70% (67.5%) and HPV-58 was the most frequently detected. HIV infection was a risk factor strongly associated with cervical infection with any HPV [adjusted Odds ratio (aOR): 17.4], multiple types of HPV (aOR: 8.9), HR-HPV (aOR: 13.2) and cervical infection with multiple HR-HPV (aOR: 8.4).

**Conclusion:**

These observations highlight the unsuspected high burden of cervical HR-HPV infection in Chadian women, and point the potential risk of further development of HPV-associated cervical precancerous and neoplastic lesions in a large proportion of women in Chad. The high rate of preventable Gardasil-9 vaccine genotypes constitutes the rationale for introducing primary vaccine prevention against cervical cancer in young female adolescents living in Chad.

## Introduction

Human papillomavirus (HPV) infection is the most common viral sexually transmitted infection (STI) worldwide and high-risk (HR)-HPV genotypes are responsible for 5.2% of all cancers worldwide, 2.2% of cancers in developed countries and 7.7% of all cancers in developing countries [1–3]. In sub-Saharan Africa, cervical cancer associated with persistent cervical HR-HPV infection is the most common cancer in women in many countries, with more than 75,000 new cases and nearly 50,000 deaths registered each year [4, 5]. According to the World Health Organization (WHO), cervical cancer will kill annually more than 443,000 women around the world by 2030 and 98% of these deaths will occur in developing countries and especially in Sub-Saharan Africa where HIV epidemic and other risk-factors are aggravating the burden of this cancer [5, 6]. Thus, cervical cancer has become progressively one of the main public health challenges to overcome in sub-Saharan Africa since the recent decades [7].

Although all HR-HPV genotypes have oncogenic potency, some of them are more frequently involved in cancers than others. These include HPV-16 and HPV-18, accounting for about 70% of all cervical cancers worldwide, and HPV-31, HPV-33, HPV-45, HPV-52, HPV-58 and HPV-68 which are among the ten most frequently isolated HR-HPV genotypes from cervical tumor biopsies [8, 9, 10]. The current 9-valent Gardasil-9 vaccine (Merck & Co. Inc., New Jersey, USA), targeting the seven primarily isolated HR-HPV genotypes from cervical cancers (HPV-16,—18, -31, -33, -45, -52 and -58) and the two primarily isolated low-risk (LR)-HPV (HPV-6 and HPV-11), has been developed according to these epidemiological data [10]. Thus, primary prevention of cervical cancer by vaccinating young girls at 10 to 14 years of age with Gardasil-9 vaccine prior sexual onset would protect them theoretically against most of the cervical HR-HPV infections [11, 12]. This primary prevention, combined with other prevention strategies (secondary and tertiary prevention) already in place in sub-Saharan Africa would overcome the high burden of cervical cancer in this continent [7, 13].

Before implementing vaccination with Gardasil-9 vaccine in African countries, it is essential to establish the epidemiological distribution of the main circulating genotypes in the general population. Indeed, these indications are critical because they allow evaluating the preventive efficiency of vaccination against HPV with the current HPV vaccine in specific populations. A wide heterogeneity in the distribution of circulating HR-HPV genotypes exists from one country to another and also between two regions within the same country throughout the African continent [14]. Generally, the epidemiological landscape of HPV infection in Sub-Saharan Africa is mostly dominated by HR-HPV genotypes targeted by the Gardasil-9 vaccine [14–20]. Some countries in sub-Saharan Africa where a good match between prevalent HPV types and Gardasil-9 has been demonstrated are now implementing vaccination of adolescents, with support from international donors [7, 21].

In Chad, a country of around 15 million people, including more than 3 million women aged more than 25 year-old [22, 23], no significant progress has been realized until now in cervical cancer prevention [24, 25]. Only one pilot study assessing the feasibility of the cytology-based screening for cervical lesions in HIV-infected women has been conducted in Chad [25]. Thus, HIV-infected Chadian women were at high-risk for low and high-grade cervical lesions, suggesting indirectly a high and unsuspected burden of cervical HPV infection in Chad [25]. In addition, primary prevention by prophylactic vaccine could likely constitute one of the best options for many women in Chad, who will likely not have the luxury of diagnostic exams and care. Unfortunately, epidemiological data on the distribution of circulating HR-HPV genotypes in the general population in Chad are lacking [26, 27]. Herein, we designed and carried out a cross-sectional study to assess the prevalence and genotypes distribution of cervical HPV infection and associated risk factors in adult women living in N’Djamena, the capital city of Chad.

## Material and methods

### Study design

The study was a descriptive, quantitative, population-based, cross-sectional survey, using a face-to-face questionnaire to collect data in 2018 among adult women living in N’Djamena, Chad.

The laboratory protocol of this study was been deposited in protocols.io website and is available at: dx.doi.org/10.17504/protocols.io.wgefbte.

### Enrolment and selection criteria

**Fig 1:**
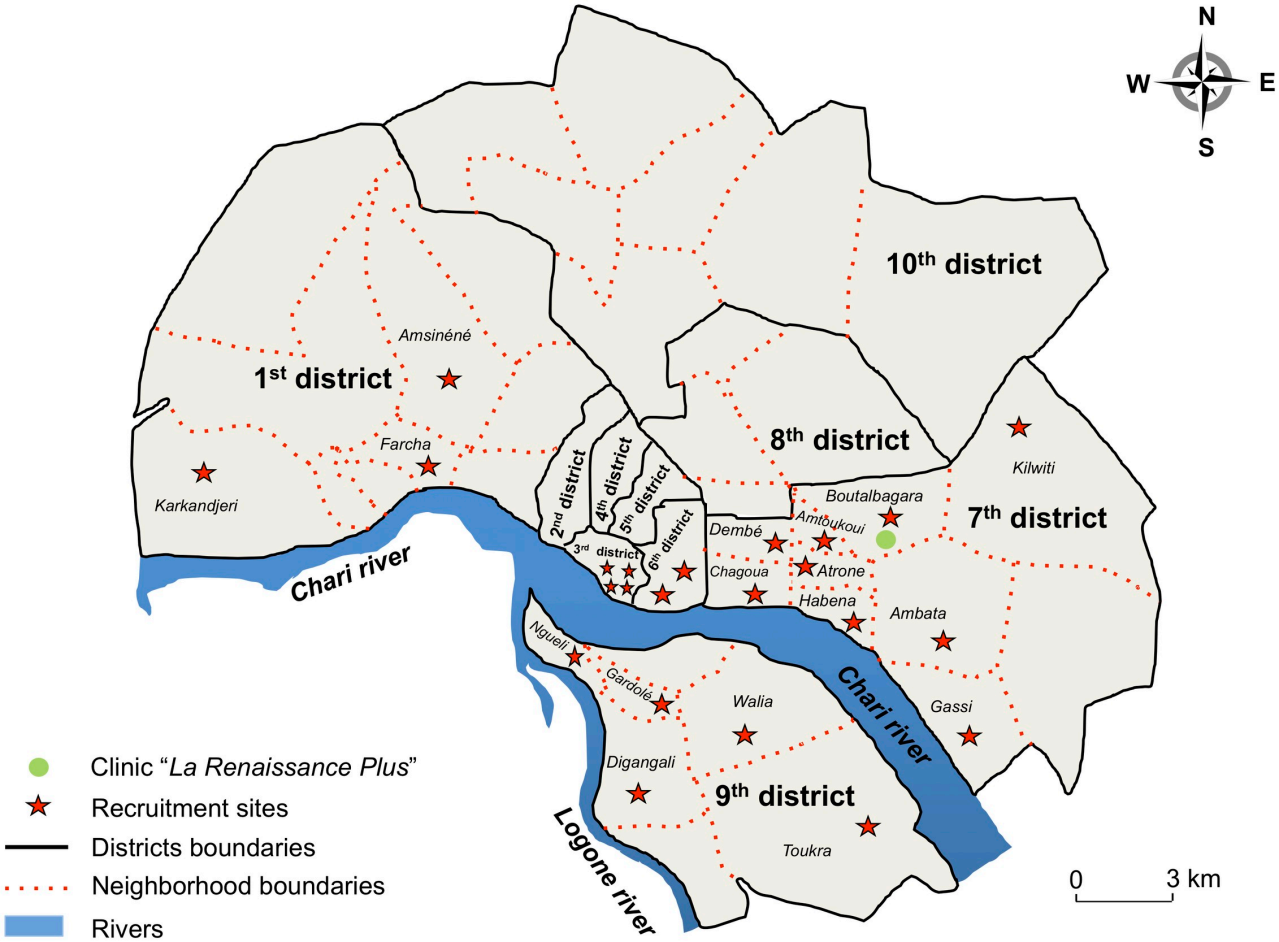
Map showing the location of 23 inclusion study sites in N’Djamena, Chad, including 5 out of 10 districts randomly selected in which neighborhoods were further chosen for awareness of the study [1^st^ district: Farcha, Amsinéné, Karkandjeri; 3^rd^ district: Gardolé, Ardep Djoumal, Kabalaye, Sabangali; 6^th^ district: Moursal, Paris-Congo; 7^th^ district: Ambata, Amtoukoui, Atrone, Boutalbagara, Chagoua, Dembé, Gassi, Habena, Kilwiti; 9^th^ district: Digagali, Gardolé, Ngueli, Tourka, Wali]. In each inclusion site, peer educators contacted adult women in community-churches or women associations during a one-month period and proposed them to be included in the study after an oral explanation and collective awareness sessions on the objectives of the survey, mainly focused on information on cervical cancer.

The capital city N’Djamena comprises 10 districts, which include a variable number of neighborhoods. Twenty-three sites in neighborhoods of 5 out of the 10 districts randomly selected were further chosen for study inclusion, as depicted in the Fig 1. In each inclusion site, peer educators contacted adult women in community-churches or women association networks during a one-month period and proposed that they participate in the study after an oral explanation and collective awareness sessions on the objectives of the survey, mainly focused on sensitization on cervical cancer and prevention strategies against cervical HPV acquisition. After oral consent, the selected women were invited, with paid transportation, to come to the clinic “*La Renaissance Plus*”, N’Djamena, which is one of the main settings for women’ sexual health in Chad, and to participate in the study. Adult women living in N’Djamena regularly attend the clinic “*La Renaissance Plus”* for gynecological examinations and for obstetrical services.

The inclusion criteria were agreeing to participate in the study, having given signed informed consent, being aged between 25–65 years, being sexually active, having no genital troubles at physical examination and having completed the questionnaire. Exclusion criteria included age less than 25 year-old and more than 65 year-old and not willing to participate to the study or to answer the face-to-face questionnaire to collect data.

After signed the informed consent form, the selected women benefited from free HIV and hepatitis B (HBV) and C (HCV) testing, by multiplex HIV/HCV/HBsAg immunochromatographic rapid test (Biosynex, Strasbourg, France) [28], clinical services including gynecological examination, family planning counseling, STIs diagnosis, laboratory analysis where necessary and appropriate treatment for those suffering from gynecologic disorders or from genital or HIV infections. All women received an information session on HIV and STIs, and completed a face-to-face questionnaire.

At inclusion, a standardized interview was conducted at the clinic “*La Renaissance Plus*”, by experienced counsellors using a face-to-face questionnaire to collect socio-demographic characteristics and behavioral data, including age, marital status, social occupation, education level, residence location in N’Djamena, history of STI, HIV status and also sexual behavioral characteristics such as the number of lifetime sexual partners and the age at first sexual intercourse.

### Samples and processing

After completing the socio-demographic data collection questionnaire, a nurse performed cervicovaginal sampling using a flocked swab (Copan Diagnostic Inc., California, USA). Briefly, specimens for molecular testing were obtained by inserting the swab into the vaginal canal until the cervix mucosa, gently rotating 5 times and then removed and immediately placed into its container and frozen at -80°C before DNA extraction procedure. Finally, cervicovaginal swab were transported in frozen ice packs, to the virology laboratory of the *hôpital Européen Georges Pompidou*, Paris, France, for molecular analyses.

### HPV detection and genotyping

DNA was extracted from the cervical swab specimen using the DNeasyBlood and Tissue kit (Qiagen, Hilden, Germany) as recommended by the manufacturer. After extraction, DNA was concentrated and eluted in 100 to 200μL of the kit elution buffer before genotyping.

The detection and the genotyping of HPV were carried out using the real-time PCR assay Anyplex II HPV28 (Seegene, Seoul, South Korea), [29]. According to the HPV classification nomenclature provided by the International Agency for Research on Cancer (IARC) [30], Anyplex II HPV28 allows to distinguish 28 HPV genotypes, including 13 high-risk types (HR-HPV -16, -18, -31, -33, -35, -39, -45, -51, -52, -56, -58, -59, and -68), 9 low-risk (LR) types (LR-HPV -6, -11, -40, -42, -43, -44,-53, -54 and -70) and then, 6 genotypes classified as probably carcinogenic (HPV-26, -61, -66, -69, -73 and -82). Briefly, 5μL of swab-extracted DNA are added into two reaction mixtures (20 μL) containing each other, one of the primers sets A and B [29]. The DNA amplification and the genotyping process are carried out in 2 reactions performed on the CFX96 real-time PCR instrument (Bio-Rad, Marnes-la-Coquette, France) [29]. The Anyplex II HPV28 genotyping test has been found to be suitable for HPV detection and genotyping in cervical secretions [29, 31–34]. Data recording and interpretation were automated with Seegene viewer software (Seegene), according to the manufacturer’s instructions. A swab sample was considered positive for any HPV if containing any of the 28 types targeted by the Anyplex II HPV28 detection test; positive for multiple HPV when containing at least 2 types of the 28 HPV types included in genotypic test; HR-HPV positive and multiple HR-HPV positive when containing respectively at least 1 HR-HPV type and at least 2 high-risk types belonging to the 19 high-risk types targeted by the Anyplex II HPV28 molecular test, irrespective of the presence of LR-HPV. The virology laboratory was accredited in 2013 by the *Comité Français d’Accréditation* (COFRAC) according to the ISO 15189 Norma for the biological markers "HPV detection" and "HPV genotyping".

### Statistical analyses

Data was entered into an Excel database and analyzed using IBM SPSS Statistics 20 software (IBM, SPSS Inc, Armonk, New York, USA). Means and standard deviations (SD) were calculated for quantitative variables and proportions for categorical variables. The results were presented as a 95% confidence interval (CI) using the Wilson score bounds. P-values (*P*) were calculated using Pearson’s χ^2^ test or Fisher’s exact test for categorical variables and the non-parametric Mann-Whitney *U*-test for quantitative variables. Logistic regression models using univariate and multivariate analyses were performed to determine the association of each independent variable [*i*.*e*., “age at inclusion”, “HIV infection”, “marital status”, “social occupation”, “education level”, “number of lifetime sexual partners” and “age at first sexual intercourse”] with the HPV type-specific cervical infections (*i*.*e*., genital infection by any type of HPV, multiple types of HPV, HR-HPV and multiple HR-HPV). All variables statistically significant (*P* < 0.05) in univariate analysis were computed into a multivariate logistic regression analysis. Crude Odds ratio (cOR) and adjusted Odds ratio (aOR) were calculated, as appropriate along with their 95% CI. Finally, a risk factor was defined as an independent variable giving in univariate analysis a cOR (along with its 95% CI) strictly higher than “1” with a *P*-value lower than 0.05. An aOR (along with its 95% CI) strictly higher than “1” with a *P*-value lower than 0.05 defines a risk factor in multivariate analysis. A protective factor was defined as an independent variable for which the cOR (along with its 95% CI) is strictly lower than “1” with a *P*-value lower than 0.05 in univariate analysis. An aOR (along with its 95% CI) strictly lower than “1” with a P-value lower than 0.05 characterizes a protective factor in multivariate analysis [35].

### Ethics statement

The study was formally approved by the Scientific Committee of the Faculty of Health Sciences of the University of N’Djamena, constituting the National Ethical Committee. All included women gave their informed signed consent to participate to the study. For each included woman, the record of the consent to participate to the study was documented on each questionnaire. This consent procedure was formally approved by the Ethical Committee.

All individual results of HPV detection and genotyping were given to each study participant, and women harboring cervical HR-HPV were further referred for diagnosis of HPV-related lesions and care at the clinic “*La Renaissance Plus*”. Furthermore, the study results have been *in extenso* reported to health authorities of Chad during the national congress of gynecologists and midwives, held from the 13^th^ to 17^th^ of November 2018 in the *Centre d’Etudes et de Formation pour le Développement* (CEFOD), N’Djamena, Chad.

## Results

### Characteristics of study population

From June to August 2018, 260 women from the 23 inclusion sites participated to the study [1^st^ district (32, 12.4%): Farcha (n = 14), Amsinéné (n = 8), Karkandjeri (n = 10); 3^rd^ district (54, 20.2%): Gardolé (n = 12), Ardep Djoumal (n = 19), Kabalaye (n = 10), Sabangali (n = 13); 6^th^ district (47, 18.3%): Moursal (n = 23), Paris-Congo (n = 24); 7^th^ district (61, 23.6%): Ambata (n = 5), Amtoukoui (n = 6), Atrone (n = 9), Boutalbagara (n = 5), Chagoua (n = 7), Dembé (n = 8), Gassi (n = 9), Habena (n = 9), Kilwiti (n = 3); 9^th^ district (66, 25.5%): Digangali (n = 10), Gardolé (n = 7), Ngueli (n = 11), Tourka (n = 12), Walia (n = 26)].

After physical examination, 7 women were excluded because of genital troubles (vaginal discharge: 2; suspicion of STI: 2; genital bleeding: 3). Finally, a total of 253 women (mean age, 35.0 years; range, 25–65) referred to the clinic “*La Renaissance Plus”* were consecutively and prospectively included in the study and their socio-demographic, sexual behavior, clinical and biological characteristics are summarized in the Table 1.

**Table 1:** Baseline characteristics of 253 adult women living in N’Djamena, in Chad.

	Study women (N = 253)
**Characteristics**	n (%) [95% CI]*
**Age**	
All age [mean (SD), years]	35.0 (9.9) (25–65 years)
25–29	80 (31.6) [25.9–37.4]
30–39	66 (26.1) [20.7–31.5]
40–49	69 (27.3) [21.7–32.7]
≥ 50	38 (15.1) [10.6–19.4]
HIV infection	9 (3.5) [1.3–5.8]
HBV infection	19 (7.5%) [4.3–10.8]
HCV infection	8 (3.2%) [1.1–5.3]
Past history of STI	10 (3.9) [1.5–6.3]
**Marital status**	
Single	15 (5.9) [3.1–8.8]
Living in couple	198 (78.3) [73.2–83.3]
Divorced	28 (11.1) [7.2–14.9]
Widow	10 (3.9) [1.5–6.3]
**Occupation**	
Student	36 (14.2) [9.9–18.5]
Unemployed	137 (54.2) [48.1–60.3]
Employed	78 (30.8) [25.2–36.5]
**Education level**	
Never schooled	50 (19.7) [14.8–24.6]
Elementary school	45 (17.8) [13.1–22.5]
High school	81 (32.1) [26.3–37.7]
University	77 (30.4) [24.7–36.1]
**Sexual behaviors**	
**Age at sexual onset** [years]	
< 16	76 (30.1) [24.4–35.7]
16–20	142 (56.2) [50.1–62.3]
> 20	35 (13.8) [9.6–18.1]
**Number of sexual partners in the life**	
One regular partner	208 (82.2) [77.5–86.9]
Several partners [1 to 5]	45 (17.8) [13.1–22.5]
**HPV DNA detection and types**	
HPV DNA in swab	58 (22.9) [17.7–28.1]
Multiple types of any HPV	16 (27.6) [16.1–39.1]
LR-HPV	25 (43.1) [30.4–55.8]
Probably oncogenic HPV	9 (15.5) [6.2–24.8]
HR-HPV	40 (68.9) [57.1–80.8]
Multiple types of HR-HPV among HR-HPV-positive swabs	10 (25.0) [11.6–38.4]
HPV-16	5 (8.6) [1.4–15.8]
HPV-18	4 (6.9) [0.4–13.4]
Any 4-valent vaccine types** among HPV-positive swabs	12 (20.7) [10.3–31.1]
Multiple 4-valent vaccine types among HPV-positive swabs	1 (1.7) [0.0–5.1]
Any 9-valent vaccine types*** among HPV-positive swabs	29 (50.0) [37.1–62.9]
Multiple 9-valent vaccine types among HPV-positive swabs	6 (10.3) [2.5–18.2]
9-valent vaccine HR-HPV types among HR-HPV-positive swabs	27 (67.5) [52.9–82.1]
9-valent vaccine HR-HPV types only among HR-HPV-positive swabs	20 (50.0) [34.5–65.5]
Non-vaccine HR-HPV types only among HR-HPV-positive swabs	13 (32.5) [17.9–47.1]
Both non-vaccine and 9-valent vaccine HR-HPV types	6 (10.3) [2.5–18.2]

*The frequency of each variable is presented with their 95% confidence interval in brackets;

**The 4-valent Gardasil-4 vaccine (Merck & Co. Inc., New Jersey, USA) is effective against HPV genotypes 6, 11, 16 and 18;

***The 9-valent Gardasil-9 vaccine (Merck & Co. Inc.) is effective against HPV genotypes 6, 11, 16, 18, 31, 33, 45, 52 and 58.

Nota bene: HPV DNA percentage was calculated among all the 253 study women; multiple types of HR-HPV positivity was calculated among swabs positive for HR-HPV DNA; the remaining HPV outcomes were calculated among swabs positive for HPV DNA.

95% CI: 95% confidence interval; HBV: Hepatitis B virus; HCV: Hepatitis C virus; HIV: Human immunodeficiency virus; STI: Sexual transmitted infection; HPV: Human papillomavirus; LR-HPV: Low-risk human papillomavirus; HR-HPV: High-risk human papillomavirus.

Using multiplex HIV/HCV/HBsAg rapid test, 9 study women (3.5%; 95% CI: 1.3–5.8) were diagnosed infected by HIV-1, 19 (7.5%; 95% CI: 4.3–10.8) by HBV (positivity for HBsAg) and 8 (3.2%; 95% CI:1.1–5.3) were seropositive for HCV.

Several women (31.6%; 95% CI: 25.9–37.4) were young, aged from 25 to 29 years, engaged in life couple with a male partner (78.3%; 95% CI: 73.2–83.3), with a relatively high education level (32.1%; 95% CI: 26.3–37.7 and 30.4%; 95% CI: 24.7–36.1, in high school level and university, respectively); but most of them were unemployed (54.2%; 95% CI: 48.1–60.3). The majority of study women (83.8%; 95% CI: 79.3–88.4) reported having only one regular sexual partner in their life, while about 10% reported to have up to 5 different sexual partners. Generally, women included in this study became sexually active at 16 to 20 years of age (56.2%; 95% CI: 50.1–62.3), whereas some of them (30.1%; 95% CI: 24.4–35.7) started their sexual life earlier, before 16 years. Finally, none of the women included in this study had ever been screened for cervical cancer and nor vaccinated against HPV infection.

### Prevalence of HPV detection and genotype distribution

**Fig 2:**
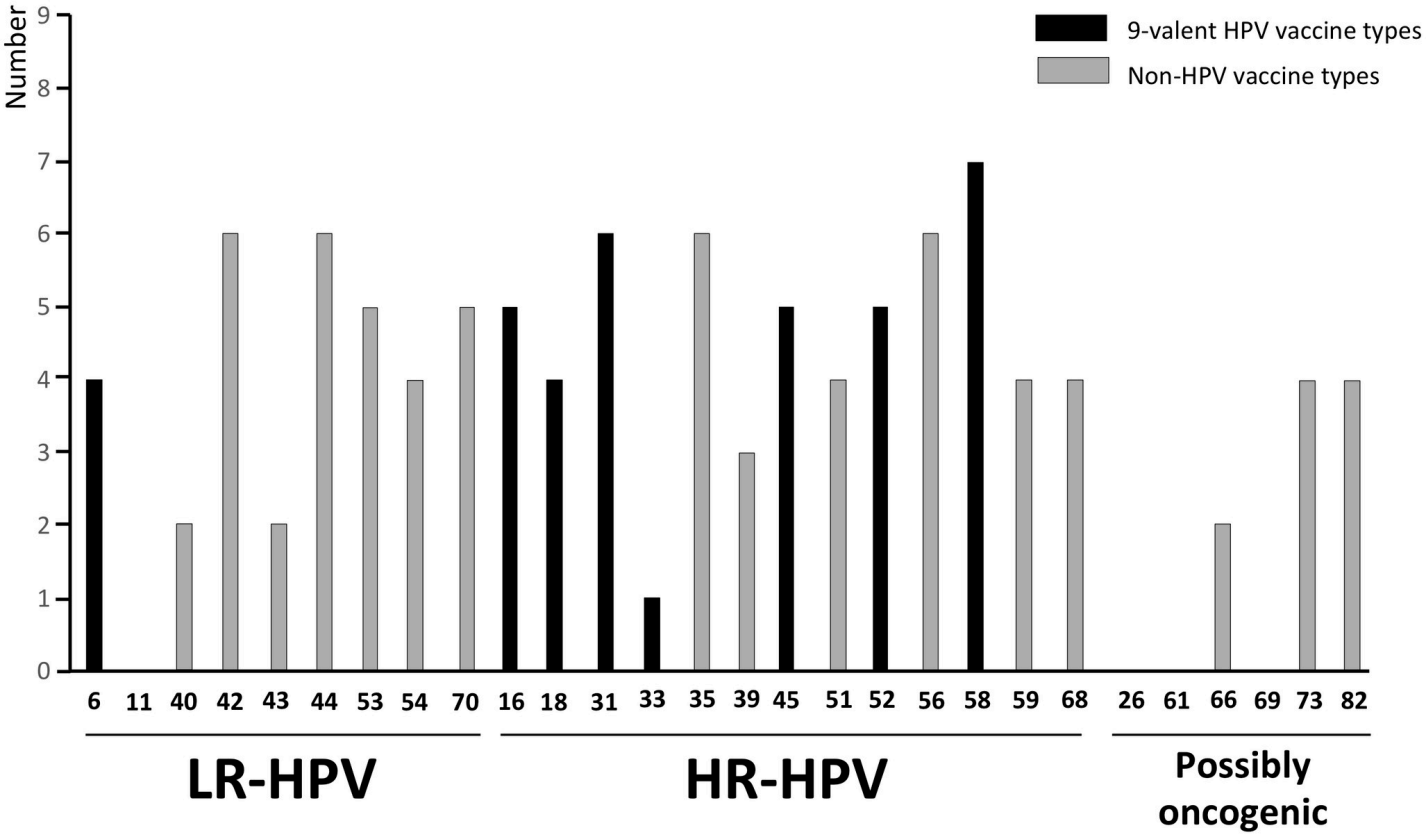
Distribution of HPV genotypes according to their inclusion in the 9-valent Gardasil-9 vaccine. Number of low-risk (LR) and high-risk (HR) HPV genotypes in 57 cervical samples positive for HPV DNA by molecular biology according to their possible prevention by 9-valent HPV vaccine among adult women (n = 253) living in N’Djamena, Chad. Nota bene. The 9-valent Gardasil-9 vaccine (Merck & Co. Inc., New Jersey, USA) is effective against HPV genotypes 6, 11, 16, 18, 31, 33, 45, 52 and 58.

Overall, 58 adult women were positive for genital HPV DNA giving a total HPV prevalence of 22.9% (95% CI: 17.7–28.1). Of those who were HPV DNA positive, 68.9% (40/58; 95% CI: 57.1–80.8) harbored cervical HR-HPV infection, providing an overall HR-HPV prevalence of 15.8% (95% CI: 11.3–20.3), as shown in the Table 1. Genital infections with multiple HPV genotypes were frequent in study women (27.6%; 95% CI: 16.1–39.1). Furthermore, 25.0% (95% CI: 11.6–38.4) of HR-HPV positive specimens contained multiple HR-HPV genotypes with an average of 2.3 HR-HPV (range, 1 to 5) per cervical swab sample. The whole distribution of HPV genotypes in HPV-DNA positive cervical samples is detailed in the Fig 2.

The Gardasil-9 vaccine HR-HPV type 58 was the predominant genotype (7/58; 12.1%), followed by the HR-HPV types 31, 35 and 56 and the LR-HPV types 42 and 44 with a prevalence of 10.3% (6/58). The 9-valent vaccine HR-HPV types 16, 45 and 52 and also the LR-HPV types 53 and 70 were present with a prevalence of 8.6% (5/58). These HPV genotypes were followed by the HR-HPV types 18, 51, 59 and 68, the LR-HPV types 6 and 54 and the probably oncogenic HPV types 73 and 82 with a prevalence of 6.9% (4/58). The HR-HPV-39 was present only in 3 women (5.2%) and a minority of study women were infected with LR-HPV types 40 and 43 (3.4%; 2/58) and HR-HPV-33 (1.7%; 1/58). Finally, none of the HPV positive samples was simultaneously positive for HPV-16 and HPV-18 (Fig 2).

### Cervical HPV DNA according to socio-demographic and behavioral characteristics and HIV serostatus

**Fig 3:**
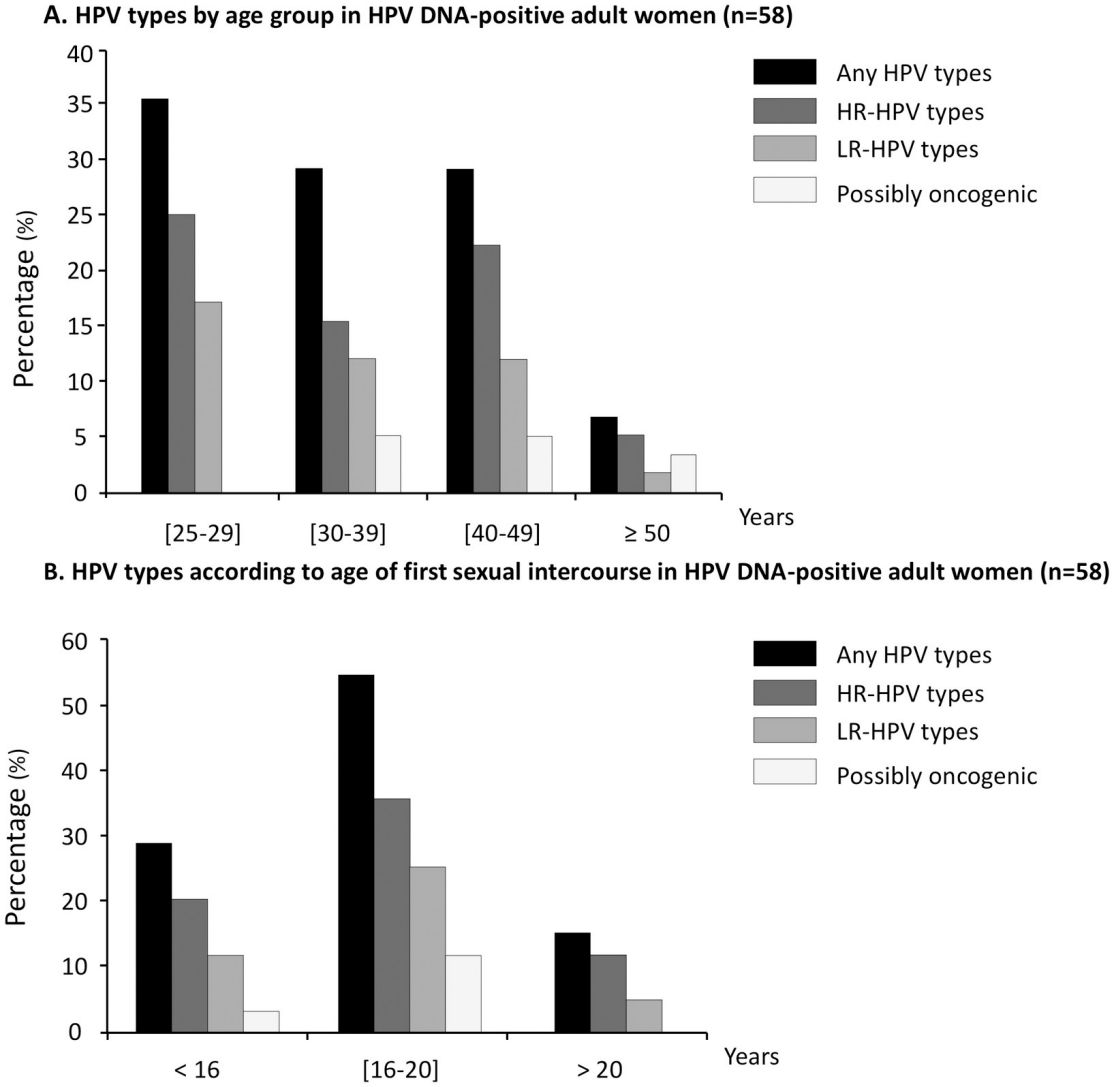
Distribution of HPV types in 57 adult women. **A.** According to age groups; **B.** According to age at first sexual intercourse.

Concerning the distribution of cervical HPV type-specific prevalence according to age groups, there was no significant difference between each of the 4 age groups. Even so, women aged from 25 to 29 years were most infected with any HPV types and LR-HPV types than both the 3 others age groups (Fig 3A). Likewise, women aged from 25 to 29 years and even those aged from 40 to 49 years harbored higher HR-HPV prevalence than women belonging to the age groups 30 to 39 years and those being 50 years of age and older. HPV types considered as probably carcinogenic were only present in women of 30 years of age and over (Fig 3A).

The distribution of cervical HPV type-specific prevalence according to the age of sexual intercourse onset is depicted in the Fig 3B. Women who had their first sexual intercourse between 16 to 20 years of age carried higher cervical HPV infection with any genotype (54.3%) compared to women who began their sexual onset below 16 years of age (29.8%) and women who started sexual intercourse after 20 years (15.8%), (*P* = 0.00006). High-risk types was similarly distributed with higher rates (36.2%) in women who started sexual intercourse between 16 to 20 years of age compared to women who had their sexual debut below 16 years (20.7%) and those who began after 20 years of age (12.1%), (*P* = 0.007).

The impact of HIV in HPV type-specific infection in the study was assessed. Seven out of the 9 HIV-infected women (77.8%) were positive for cervical HPV DNA and 85.7% (6/7) of these infections were caused by HR-HPV genotypes, with the predominant genotype being HPV-56 (42.8%; 3/7). Moreover, when compared to HIV-negative, HIV-positive women were significantly more infected with any type of HPV (77.8%; 95% CI: 50.6–100.0 *versus* 20.5%; 95% CI: 15.4–25.6; *P =* 0.0005), HR-HPV types (66.7%; 95% CI: 35.8–97.5 *versus* 13.5%; 95% CI: 9.2–17.8; *P =* 0.0005), multiple infections with any type of HPV (33.3%; 95% CI: 2.5–64.1 *versus* 4.9%; 95% CI: 2.2–7.6; *P =* 0.01) and multiple HR-HPV types (22.2%; 95% CI: 0.0–49.4 *versus* 3.7%; 95% CI: 1.3–6.1; *P =* 0.03) (Table 2).

**Table 2:** Logistic regression analyses for cervical HPV-associated risk factors in 253 adult women living in N’Djamena, Chad.

	Any HPV (N = 58)					Multiple HPV (N = 16)					HR-HPV (N = 40)					Multiple HR-HPV (N = 10)				
Risk factors	n (%)	cOR (95%CI)	P*	aOR (95%CI)	*P*	n (%)	cOR (95%CI)	P*	aOR (95%CI)	*P*	n (%)	cOR (95%CI)	P*	aOR (95%CI)	*P*	n (%)	cOR (95%CI)	P*	aOR (95%CI)	*P*
**Age** (years)																				
25–29	20 (34.5)	Reference	-	NA**	NA	6 (37.5)	Reference	-	NA	NA	15 (37.5)	Reference	-	NA	NA	3 (30.0)	Reference	-	NA	NA
30–39	17 (29.3)	1.2 (0.6–2.4)	0.071	NA	NA	3 (18.8)	0.6 (0.2–2.3)	0.769	NA	NA	9 (22.5)	0.8 (0.4–1.8)	0.573	NA	NA	3 (30.0)	1.2 (0.3–4.9)	0.724	NA	NA
40–49	17 (29.3)	1.1 (0.6–2.2)	0.087	NA	NA	5 (31.2)	1.2 (0.4–3.7)	0.773	NA	NA	13 (32.5)	1.4 (0.7–2.8)	0.418	NA	NA	3 (30.0)	1.1 (0.3–4.6)	1.00	NA	NA
≥ 50	4 (6.9)	0.4 (0.08–1.08)	0.057	NA	NA	2 (12.5)	0.8 (0.2–3.7)	0.771	NA	NA	3 (7.5)	0.4 (0.1–1.4)	0.147	NA	NA	1 (10.0)	0.6 (0.1–5.0)	1.00	NA	NA
**HIV infection**																				
No	51 (87.9)	Reference	-	Reference	-	13 (81.2)	Reference	-	Reference	-	34 (85.0)	Reference	-	Reference	-	8 (80.0)	Reference	-	Reference	-
Yes	7 (12.1)	13.2 (2.7–65.7)	0.001	17.4 (3.2–94.9)	0.001	3 (18.8)	8.9 (2.0–39.6)	0.014	8.9 (2.0–39.6)	0.004	6 (15.0)	12.4 (2.9–51.7)	0.001	13.2 (3.1–56.6)	< 0.001	2 (20.0)	8.4 (1.5–47.2)	0.044	8.4 (1.5–47.2)	0.015
**Marital status**																				
Single	4 (6.9)	1.2 (0.4–4.0)	0.753	NA	NA	1 (6.2)	1.1 (0.1–8.6)	0.955	NA	NA	2 (5.0)	0.8 (0.2–3.7)	1.00	NA	NA	1 (10.0)	1.8 (0.2–15.4)	0.464	NA	NA
Living in couple	44 (75.9)	Reference	-	NA	NA	14 (87.5)	Reference	-	NA	NA	33 (82.5)	Reference	-	NA	NA	8 (80.0)	Reference	-	NA	NA
Divorced	2 (3.4)	0.8 (0.08–4.5)	0.822	NA	NA	0 (0.0)	NA	1.00	NA	NA	1 (2.5)	0.6 (0.1–4.7)	1.00	NA	NA	0 (0.0)	NA	1.00	NA	NA
Widowed	8 (13.8)	1.4 (0.6–3.4)	0.451	NA	NA	1 (6.2)	0.5 (0.1–4.1)	1.00	NA	NA	4 (10.0)	0.9 (0.3–2.7)	1.00	NA	NA	1 (10.0)	0.9 (0.1–7.3)	1.00	NA	NA
**Occupation**																				
Unemployed	29 (50.0)	Reference	-	Reference	-	9 (56.2)	Reference	-	NA	NA	21 (52.5)	Reference	-	1.5 (0.1–28.9)	0.805	6 (60.0)	Reference	-	NA	NA
Student	13 (22.4)	2.2 (1.1–4.6)	0.042	3.2 (0.2–42.9)	0.381	5 (31.2)	3.0 (0.98–9.3)	0.059	NA	NA	10 (25.0)	2.4 (1.1–5.5)	0.034	3.5 (0.2–66.6)	0.411	3 (30.0)	2.7 (0.7–11.1)	0.145	NA	NA
Employed	16 (27.6)	0.8 (0.4–1.6)	0.542	1.45 (0.1–17.8)	0.761	2 (12.5)	0.3 (0.1–1.4)	0.160	NA	NA	9 (22.5)	0.6 (0.3–1.3)	0.214	3.4 (0.19–59.9)	0.376	1 (10.0)	0.2 (0.03–1.9)	0.182	NA	NA
**Education level**																				
Never schooled	10 (17.2)	0.8 (0.4–1.7)	0.780	NA	NA	2 (12.5)	0.5 (0.1–2.5)	0.815	NA	NA	6 (15.0)	0.7 (0.3–1.7)	0.742	NA	NA	2 (20.0)	1.02 (0.2–4.9)	0.960	NA	NA
Elementary	12 (20.7)	1.3 (0.6–2.7)	0.510	NA	NA	3 (18.8)	1.1 (0.2–3.9)	0.917	NA	NA	8 (20.0)	1.2 (0.5–2.8)	0.690	NA	NA	1 (10.0)	0.5 (0.1–4.1)	1.00	NA	NA
High school	16 (27.6)	0.8 (0.4–1.5)	0.410	NA	NA	5 (31.2)	1 (0.3–2.9)	0.946	NA	NA	10 (25.0)	0.7 (0.3–1.4)	0.300	NA	NA	3 (30.0)	0.9 (0.2–3.6)	1.00	NA	NA
University	20 (34.5)	Reference	-	NA	NA	6 (37.5)	Reference	-	NA	NA	16 (40.0)	Reference	-	NA	NA	4 (40.0)	Reference	0.498	NA	NA
**Numbers of sexual partners in life**																				
One regular partner	52 (89.7)	Reference	-	NA	NA	14 (87.5)	Reference	-	NA	NA	34 (85.0)	Reference	-	NA	NA	9 (90.0)	Reference	-	NA	NA
Several partners [1 to 5]	6 (10.3)	1.9 (0.8–4.8)	0.168	NA	NA	2 (12.5)	1.4 (0.3–6.3)	1.00	NA	NA	6 (15.0)	1.1 (0.4–2.9)	0.822	NA	NA	1 (10.0)	1.8 (0.2–14.4)	1.00	NA	NA
**Age at sexual onset** (years)																				
< 16	17 (29.3)	0.9 (0.5–1.8)	0.890	NA	NA	3 (18.8)	0.5 (0.1–1.9)	0.405	NA	NA	12 (30.0)	1.0 (0.5–2.1)	0.995	NA	NA	2 (20.0)	0.6 (0.1–2.7)	0.728	NA	NA
16–20	32 (55.2)	Reference	-	NA	NA	10 (62.5)	Reference	-	NA	NA	21 (52.5)	Reference	-	NA	NA	5 (50.0)	Reference	-	NA	NA
> 20	9 (15.5)	1.2 (0.5–2.7)	0.672	NA	NA	3 (18.8)	1.5 (0.4–5.5)	0.471	NA	NA	7 (17.5)	1.4 (0.6–3.5)	0.464	NA	NA	3 (30.0)	2.8 (0.7–11.5)	0.147	NA	NA

*The P-value is calculated using Pearson’s χ2 test or Fisher’s exact test for categorical variables;

**Not attributable (“NA”)" corresponds to variables giving crude Odds ratio not significant in univariate analysis (P > 0.05) and consequently not taken into account in multivariate analysis.

aOR: adjusted Odds ratio; cOR: crude Odds ratio; HIV: Human immunodeficiency virus; HR-HPV: High-risk human papillomavirus; LR-HPV: Low-risk human papillomavirus; NA: Not attributable; n: Number (size of study group); CI: Confidence interval.

### Predictive risk factors for cervical HPV shedding by logistic regression analyses

The associations between each one of the type-specific HPV infections with their potential predictive risk factors were assessed by logistic regression analysis, as shown in the Table 2.

In univariate analysis, the variable “HIV infection” was significantly associated with cervical infection with any type of HPV (cOR: 13.2, 95% CI: 2.7–65.7%; *P* = 0.001), multiple types of HPV (cOR: 8.9, 95% CI: 2.0–39.6*; P* = 0.014), HR-HPV (cOR: 12.4, 95% CI: 2.9–51.7%; P = 0.001) and cervical infection with multiple HR-HPV (cOR: 8.4, 95% CI: 1.5–47.2%; *P* = 0.044). Likewise, the variable “being student” was statistically associated with the cervical carriage of any type of HPV (cOR: 2.2, 95% CI: 1.1–4.6%; *P* = 0.042) and HR-HPV (cOR: 2.4, 95% CI: 1.1–5.5%; *P = 0*.*034*). Another statistical association seems to emerge between the variables "being 50 years of age or older" and getting infected with any HPV (cOR: 0.4, 95% CI: 0.08–1.08%; *P* = 0.057). “Being 50 years of age or older” seems to have a slightly protective effect against cervical infection with any HPV, but the statistical power of this protective effect was canceled.

The variable “HIV infection” and the three subclasses of the variable “Occupation” (being unemployed, student or employed) were computed into the final multivariate analysis. Only the variables “HIV infection” succeeded to maintain its statistical link with the HPV outcomes variables. Indeed, HIV infection was strongly associated with cervical infection with any HPV (aOR: 17.4, 95% CI: 3.2–94.9%; *P* = 0.001), multiple types of HPV (aOR: 8.9, 95% CI: 2.0–39.6%; *P* = 0.004), HR-HPV (aOR: 13.2, 95% CI: 3.1–56.6%; *P* < 0.001) and cervical infection with multiple different HR-HPV (aOR: 8.4, 95% CI: 1.5–47.2%; *P* = 0.015). Finally, the other explicative variables taken into account in this analysis, such as “age at inclusion”, “marital status”, “education level”, “number of lifetime sexual partners” and “age at first intercourse” were found to be not significantly associated with any of the four outcomes variables characterizing cervical HPV infections in the study women.

### Possible efficiencies of cervical HPV prevention by Gardasil vaccines

Finally, possible efficiencies of cervical HPV prevention in study women by the 4- and 9-valent Gardasil vaccines were further assessed. Less than 20% (12/58) of the HPV-positive cervical samples contained one of the 4 genotypes (HPV types 6, 11, 16 and 18) covered by the Gardasil-4 vaccine and less over (1.7%) contained simultaneously several of these 4-valent vaccine-genotypes. Regarding the Gardasil-9 vaccine, 50% (29/58) of HPV-positive cervical samples harbored at least 1 HPV type prevented by the 9-valent HPV vaccine, with 17.8% (5/28) of them which contained multiple HPV genotypes (Fig 2 and Table 1). Moreover, about 70% (27/40) of study women with a cervical HR-HPV infection harbored at least one 9-valent vaccine high-risk genotype. When excluding the non-vaccine type, half (50%; 20/40) of cervical HR-HPV infections were exclusively due to a 9-valent-vaccine high-risk genotype.

## Discussion

The present study depicts for the first time the molecular epidemiology of cervical HPV infection in adult women living in N’Djamena, the capital city of Chad. One of five (22.9%) of study women had cervical HPV, which was mostly constituted of cervical HR-HPV (68.9%), 27.6% of HPV positive women harboring multiple genotypes. In this series, HIV seropositivity (3.5%) constituted the main risk factor significantly associated with an increasing risk of being infected by cervical HPV. The majority (≈70%) of women infected by cervical HR-HPV showed high-risk genotypes covered by the 9-valent Gardasil-9 vaccine, with the HR-HPV-58 being the predominant genotype, followed by HPV-31, HPV-16, HPV-45, HPV-52 and HPV-18. Remarkably, most of these high-risk genotypes (HPV-58, HPV-31, HPV-45 and HPV-52) were not covered by the Gardasil-4 vaccine. Taken together, these observations highlight the unsuspected high burden of cervical HR-HPV infection in women more than 25 years living in Chad, with a high rate of preventable Gardasil-9 vaccine genotypes. These molecular findings demonstrate that cervical HR-HPV infection and associated risk for cervical cancer involve a large proportion of adult women living in Chad. According to our findings, with the hypothesis of a rate of genital shedding of HR-HPV at 15.8%, it may be estimated that at least 460,000 Chadian women aged more than 25 years thorough the country may be at risk for cervical cancer during their life. Finally, an *a priori* good predictive efficacy of the prophylactic Gardasil-9 vaccine could be envisioned for the primary prevention of cervical HPV infection in Chad. Thus, since HPV DNA molecular screening in Chad remains currently opportunistic or unavailable for many women, prophylactic HPV vaccination using a multivalent vaccine may help in achieving cervical cancer elimination.

In this large series of never screened adult women attending the obstetrics and gynecology clinic *“La Renaissance Plus”* of N’Djamena, we observed a high frequency (68.9%) of cervical HR-HPV genotypes in HPV DNA positive women. Such high prevalence of HR-HPV appears notably higher as compared to those commonly reported in unscreened African women aged from 25 years of age and older, with cervical HR-HPV infection rates never exceeding half of the HPV-positive women. Indeed, cervical HR-HPV prevalences in adult women in sub-Saharan Africa vary frequently across regions of the same country and also from one country to another, and vary between 5.4% in Djibouti [36], 10.0% and 36.5% in Nigeria [17, 37], 12.5% in Democratic Republic of the Congo [38], 18.5% and 34.0% in Cameroon [39, 40], 19.3% in Malawi [41], 20.3% in Tanzania [42], 22.2% in Rwanda [43], 25.0% in South-Africa [19], 25.4% and 38.3% in Burkina Faso [44, 45], 39.3% in Madagascar [46] and finally 46.2% in Swaziland [47]. However, other studies conducted in other adult women living in sub-Saharan Africa reported high prevalences of cervical HR-HPV infection similar to that reported in the present series with HR-HPV prevalence, ranging from 60.4% in Nigeria [15], to 67.9% and 68.5% in South Africa [20, 48]. Only studies conducted in younger, but sexual active African women less than 25 years reported higher HR-HPV prevalences (70.0% to 84.0%) [18, 49–51] than that observed in our study. Different prevalences of cervical HPV in asymptomatic African women between studies, despite the fact that these studies were been conducted on the same continent and on quite similar populations, may be due to several factors, including firstly genital sampling methods, HPV molecular testing tools and behavioral and socio-demographic factors. On the other hand, the age could also constitute a major factor explaining the differences in HPV prevalences between studies, since the highest prevalence of HPV infection is found in the adolescence and early adulthood, soon after the sexual onset and then decline over time [7, 52].

Concerning HPV genotype distribution, women carrying HR-HPV infection showed remarkable high rates (≈70%) of high-risk types targeted by the Gardasil-9 vaccine, with HPV-58 being the most represented genotype. Moreover, five other high-risk types included in the 9-valent vaccine (HPV-31, HPV-16, HPV-45, HPV-52 and HPV-18 in decreasing order) belonged to the four most detected genotypes; HPV-16 and HPV-18 being respectively the third and the fourth most represented. In the other hand, two high-risk types were not covered by the Gardasil-9 vaccine, HPV-35 and HPV-56.

Most previous studies conducted in sub-Saharan African countries depict a wide heterogeneity in the distribution of the main HR-HPV in women [14–20, 36, 38–48]. The predominance of cervical HR-HPV-58 genotype in Chadian women who never undergone cervical screening was never reported in other series from sub-Saharan Africa, and may constitute a particularity of the molecular epidemiology of HR-HPV in women living in Chad. Our observations furthermore demonstrate the preponderance of cervical HR-HPV targeted by the Gardasil-9 vaccine in asymptomatic women living in Chad, as commonly observed in women with normal cytology and those with high-grade intraepithelial lesions in other sub-Saharan African countries [14]. These indications are of paramount importance because they allow evaluating the predictive efficiency of vaccination against HPV with the Gardasil-9 vaccine in young Chadian girls. Indeed, our observations suggest that a large majority (≈70%) of these HR-HPV infections could have been, *a priori*, prevented using the Gardasil-9 vaccine. In the context of the important population of females living in Chad, estimated in 2018 at around 7,600,000 [22], our epidemiological observations represent a powerful argument in favor of the introduction of the Gardasil-9 vaccine in the national immunization program in order to strengthen cervical cancer prevention.

In the present series, two main groups characterized by their risk factors for HPV infection were identified. Firstly, HIV-infected women were found to be 17-times more at risk for cervical infection with any type of HPV and more than 8-times for multiple types of HPV than HIV-negative women. Regarding cervical HR-HPV infection, HIV-infected women were 13-times more at risk than HIV-negative women and more than 8-times for infection with multiple high-risk types. These findings are consistent with previous reports demonstrating the aggravating effect of HIV in the burden of HR-HPV infection in women and particularly in the African settings [53–55]. Secondly, the student women, being mostly 25 to 29 year-old, were more than 2 times more at-risk for both cervical infection with any type of HPV and HR-HPV infection than women involved in other social occupations. However, this risk seems to fade in multivariate analysis. In our study, we did not find by multivariate analysis any association between any HPV and more specifically HR-HPV genital carriage and young age, although age less than 30 years was previously reported as a risk factor frequently associated with cervical HR-HPV infection in African women [15, 42, 50]. The possibility exists that our study may have lacked of power due to too small sample size of young women group to evidence such association. Other factors such as genital toilet or hygiene practice during menses which modify the cervicovaginal ecology [56, 57], or the higher economic status of student women which constitutes per se a multiple risk behavior [58], could be also envisioned.

Our observations highlight that women living in Chad constitute a neglected high risk group for cervical HR-HPV infection and consequently for cervical cancer. These findings constitute the first report ever provided on the epidemiological burden of HPV infection in Chad. The very high prevalence of cervical HR-HPV in adult women clearly demonstrates that cervical HR-HPV infection in Chad constitutes a major public health problem which remains largely unsuspected. Therefore, there is an urgent need for implementing a cervical cancer prevention program in Chad, as recommended by the WHO [59]. According to Mortier and colleagues, the cytology-based cervical cancer screening in women in Chad is feasible with low cost and easy to interpret visual technics; and could be integrated in existing healthcare structures [25]. Indeed, for these women carrying cervical HR-HPV infection, only secondary prevention with regular screening for precancerous lesions by cytology and the monitoring of the viral persistence by HPV molecular testing, remains the only alternative to prevent the disease progression into invasive cervical cancer. However, in the context of Chad, a very low-income country, there is a serious lack of pathologist specialist thereby making conventional cytology not suitable and reinforcing on the other hand the great necessity to implement HPV DNA testing with molecular technologies, including point-of-care analyzers [7]. Indeed, HPV DNA testing is an excellent alternative to cytology for cervical cancer screening, because it is higher sensitive, more reproducible and easy to interpret than cytology [7]. Moreover, the rapid turnaround time of HPV DNA testing system could promote the “see and treat” approach recommended by the WHO [59], thus allowing to maximize all the medical support in a single visit and avoiding the loss of women positive for HR-HPV. Furthermore, taking into account that most adult Chadian women are living in remote rural areas, far away from adequate healthcare structure, self-collection cervical specimen carried out at home by women themselves could also represent a relevant alternative allowing to increase the coverage of screening when coupled with low-cost HPV DNA testing technologies [7]. This model of multicomponent prevention strategy that could also integrate existing HIV healthcare structures is critical to better target adult women living in Chad at risk for cervical HR-HPV infection and related diseases. However, without medical insurance, as it is the case for most women in Chad, secondary prevention based on HPV DNA molecular diagnostic tests is still beyond the reach of most Chadian women. Consequently, along with the secondary prevention strategy adapted to Chadian adult women, around 600,000 adolescents girls aged from 10 to 14 year-old in Chad [22, 23], could benefit from a national immunization program with the current prophylactic Gardasil-9 vaccine prior their sexual life onset [7].

We tried to limit the selection bias of the study population in order to make this survey as much representative as possible of the female population in Chad. Thus, the study inclusions were initially carried out by random sampling of districts in N’Djamena, with further selection of neighborhoods inclusions site. Study women were referred to the clinic “*La Renaissance Plus”* of N’Djamena, although they were not part of the patients from the clinic, avoiding the obvious bias of recruitment by health care facilities. Furthermore, 9 (3.5%), 19 (7.5%) and 8 (3.2%) study participants were HIV-, HBsAg- and HCV-specific antibody- positive, respectively, in accordance with the high endemicity of these three major chronic viral infections in Chad [60, 61]. Indeed, Chad is a country of generalized HIV epidemic with the adult prevalence rate at 3.5% in N’Djamena [60], as observed in our series. Chad is also a country of high HBV and HCV infections prevalences in adults, and in our study the prevalences of these viral infections were of the same order as those previously reported in N’Djamena in 2014 by Bessimbaye and colleagues [61]. Consequently, the rates of these three epidemiological infectious markers observed in our series tend to reflect the general epidemiology in Chad, thereby making our study sample close to the general population in Chad. However, our study has some limitations. First, the representativeness of the included study population is not completely ensured for the other districts of N’Djamena which were not selected. Such inclusion bias could be extended in other cities and rural remote territories of Chad, where no women could be included. Second, the small sample size of our study population may have introduced a bias, although more than 250 women were enrolled. Third, participants were included on a voluntary basis. This latter approach may be a source of recruitment bias. Furthermore, by using a face-to-face questionnaire, the validity of the answers to the questions was collected from participants, including items related to the intimacy of their sexual life. In Chad, a high number of women are unable to read and write properly, rendering a self-administered questionnaire was not relevant for this reason; however, it has been documented that questions about personal privacy are best collected anonymously by means of a self-administered questionnaire [62]. In addition, the recruitment based on community-churches, and associative networks may have introduced a selection bias. Moreover, the study women said that they agreed to participate in the study because they considered that confidentiality was guaranteed at the clinic “*La Renaissance Plus* " of N’Djamena, which is considered by the public as the benchmark of good practice in the area of obstetrics and gynecology. Finally, in regard of our small sample size some risk factors may have been underestimated in the statistical analyses. Despite these possible limitations, our study constitutes the first report providing objective information on molecular epidemiology of cervical HPV in symptom-free adult women living in N’Djamena, and thus enables us to show all the interest of the prophylactic vaccination by the Gardasil-9 vaccine in eligible young adolescent women, that could be very possibly generalized to the whole country.

In conclusion, an unsuspected high prevalence of cervical HR-HPV infection, worsened by HIV, was observed in women more than 25 years living in N’Djamena, Chad. The study women recruitment according to a population-based approach allows to extrapolate our observations to the general female population, and to make the hypothesis that the burden of HR-HPV shedding may be particularly high in Chadian women, and consequently, the risk of further development of HPV-associated cervical precancerous and neoplastic lesions. In addition, the HPV genotypes distribution in genital secretions of study women indicates the probable existence of a particular molecular epidemiology of HPV in Chad, with rare genotypes being predominant such as HPV-58. Finally, our results demonstrate that the majority of cervical HR-HPV corresponds to preventable Gardasil-9 vaccine genotypes, making the rationale of introducing primary prevention against cervical cancer in young female adolescents living in Chad by prophylactic vaccination. Taken together, our findings point for the first time the unsuspected high exposure of Chadian women to oncogenic HR-HPV, making thus cervical cancer, its diagnosis and prevention, some of the most important public health challenges that Chad will faced in a near future.

## Supporting information

S1 File Baseline database of the 253 study women living in N’Djamena, Chad. Microsoft excel baseline database containing all information from the study women analyzed in the study and related to their sociodemographic and clinical characteristics and also information associated to the results of the HPV testing and genotyping.
(XLSX)

S2 File STROBE Checklist. (DOC)

## References

[1] Scheurer ME, Tortolero-Luna G, Adler-Storthz K. Human papillomavirus infection: biology, epidemiology, and prevention. Int J Gynecol Cancer. 2005;15(5):727–46. 10.1111/j.1525-1438.2005.00246.x 16174218

[2] Parkin DM. The global health burden of infection-associated cancers in the year 2002. Int J Cancer. 2006;118(12):3030–44. 10.1002/ijc.21731 16404738

[3] Mboumba Bouassa RS, Mbeko Simaleko M, Camengo SP, Mossoro-Kpinde CD, Veyer D, Matta M et al Unusual and unique distribution of anal high-risk human papillomavirus (HR-HPV) among men who have sex with men living in the Central African Republic. PLoS One. 2018;13(5):e0197845 10.1371/journal.pone.0197845 29795661 PMC5967740

[4] De Vuyst H, Alemany L, Lacey C, Chibwesha CJ, Sahasrabuddhe V, Banura C et al The burden of human papillomavirus infections and related diseases in sub-saharan Africa. Vaccine. 2013;31 Suppl 5:F32–46. 24331746 10.1016/j.vaccine.2012.07.092 PMC4144870

[5] Ferlay J, Soerjomataram I, Dikshit R, Eser S, Mathers C, Rebelo M et al Cancer incidence and mortality worldwide: sources, methods and major patterns in GLOBOCAN 2012. Int J Cancer. 2015;136(5):E359–86. 10.1002/ijc.29210 25220842

[6] World health Organization, 2015. Projections of mortality and causes of death, 2015 and 2030. (Last accessed July 2018). http://www.who.int/healthinfo/global_burden_disease/projections/en/.

[7] Mboumba Bouassa RS, Prazuck T, Lethu T, Jenabian MA, Meye JF, Bélec L. Cervical cancer in sub-Saharan Africa: a preventable noncommunicable disease. Expert Rev Anti Infect Ther. 2017;15(6):613–627. 10.1080/14787210.2017.1322902 28440679

[8] de Sanjose S, Quint WG, Alemany L, Geraets DT, Klaustermeier JE, Lloveras B et al. Human papillomavirus genotype attribution in invasive cervical cancer: a retrospective cross-sectional worldwide study. Lancet Oncol. 2010 11;11(11):1048–56. 10.1016/S1470-2045(10)70230-8 20952254

[9] Bruni L, Diaz M, Castellsagué X, Ferrer E, Bosch FX, de Sanjosé S. Cervical human papillomavirus prevalence in 5 continents: meta-analysis of 1 million women with normal cytological findings. J Infect Dis. 2010;202(12):1789–99. 10.1086/657321 21067372

[10] Joura EA, Giuliano AR, Iversen OE, Bouchard C, Mao C, Mehlsen J et al A 9-valent HPV vaccine against infection and intraepithelial neoplasia in women. N Engl J Med. 2015;372(8):711–23. 10.1056/NEJMoa1405044 25693011

[11] Zhai L, Tumban E. Gardasil-9: A global survey of projected efficacy. Antiviral Res. 2016;130:101–9. 10.1016/j.antiviral.2016.03.016 27040313

[12] Ruiz-Sternberg ÁM, Moreira ED Jr, Restrepo JA, Lazcano-Ponce E, Cabello R, Silva A et al Efficacy, immunogenicity, and safety of a 9-valent human papillomavirus vaccine in Latin American girls, boys, and young women. Papillomavirus Res. 2018;5:63–74. 10.1016/j.pvr.2017.12.004 29269325 PMC5887018

[13] Lynge E, Rygaard C, Baillet MV, Dugué PA, Sander BB, Bonde J et al Cervical cancer screening at crossroads. APMIS. 2014;122(8):667–73. 10.1111/apm.12279 25046198

[14] Ogembo RK, Gona PN, Seymour AJ, Park HS, Bain PA, Maranda L et al Prevalence of human papillomavirus genotypes among African women with normal cervical cytology and neoplasia: a systematic review and meta-analysis. PLoS One. 2015;10(4):e0122488 10.1371/journal.pone.0122488 25875167 PMC4396854

[15] Akarolo-Anthony SN, Famooto AO, Dareng EO, Olaniyan OB, Offiong R, Wheeler CM et al Age-specific prevalence of human papilloma virus infection among Nigerian women. BMC Public Health. 2014;14:656 10.1186/1471-2458-14-656 24972674 PMC4094683

[16] Manga MM, Fowotade A, Abdullahi YM, El-Nafaty AU, Adamu DB, Pindiga HU et al Epidemiological patterns of cervical human papillomavirus infection among women presenting for cervical cancer screening in North-Eastern Nigeria. Infect Agent Cancer. 2015;10:39 10.1186/s13027-015-0035-8 26435733 PMC4592568

[17] Kennedy NT, Ikechukwu D, Goddy B. Risk factors and distribution of oncogenic strains of human papillomavirus in women presenting for cervical cancer screening in Port Harcourt, Nigeria. Pan Afr Med J. 2016;23:85 10.11604/pamj.2016.23.85.8510 27222684 PMC4867190

[18] Edna Omar V, Orvalho A, Nália I, Kaliff M, Lillsunde-Larsson G, Ramqvist T et al Human papillomavirus prevalence and genotype distribution among young women and men in Maputo city, Mozambique. BMJ Open. 2017;7(7):e015653 10.1136/bmjopen-2016-015653 28716790 PMC5722086

[19] Mbatha JN, Taylor M, Kleppa E, Lillebo K, Galappaththi-Arachchige HN, Singh D et al High-risk human papillomavirus types in HIV-infected and HIV-uninfected young women in KwaZulu-Natal, South Africa: implications for vaccination. Infect Dis (Lond). 2017;49(8):601–608. 28403727 10.1080/23744235.2017.1312513

[20] Mbulawa ZZA, van Schalkwyk C, Hu NC, Meiring TL, Barnabas S, Dabee S et al High human papillomavirus (HPV) prevalence in South African adolescents and young women encourages expanded HPV vaccination campaigns. PLoS One. 2018;13(1):e0190166 10.1371/journal.pone.0190166 29293566 PMC5749739

[21] Gallagher KE, Howard N, Kabakama S, Mounier-Jack S, Burchett HED, LaMontagne DS et al Human papillomavirus (HPV) vaccine coverage achievements in low and middle-income countries 2007–2016. Papillomavirus Res. 2017;4:72–78. 10.1016/j.pvr.2017.09.001 29179873 PMC5710977

[22] Institut National de la Statistique, des Etudes Economiques et Démographiques (INSEED), Ministère de L'Économie et de la Planification du Développement. République du Tchad. TCHAD-POPULATION. (Last accessed: December 2018). http://www.inseed-td.net/index.php/thematiques/statistique-demographique/population.

[23] Institut National de la Statistique, des Études Économiques et Démographiques (INSEED), Ministère de la Santé Publique (MSP) et ICF International, 2014–2015. Enquête Démographique et de Santé et à Indicateurs Multiples (EDS-MICS 2014–2015). Rockville, Maryland, USA: INSEED Mai 2016, MSP et ICF International. (Last accessed: December 2018). https://dhsprogram.com/pubs/pdf/fr317/fr317.pdf.

[24] Alliance for Cervical Cancer Prevention. The Case for Investing in Cervical Cancer Prevention. Seattle: ACCP; 2004. Cervical Cancer Prevention Issues in Depth, No. 3. (Last accessed: July 2018). http://screening.iarc.fr/doc/RH_accp_case.pdf.

[25] Mortier E, Doudéadoum N, Némian F, Gaulier A, Kemian M. [Feasibility of cervical smear in HIV-positive women living in Chad]. Bull Soc Pathol Exot. 2016;109(3):180–4. 10.1007/s13149-016-0496-z 27299910

[26] Finocchario-Kessler S, Wexler C, Maloba M, Mabachi N, Ndikum-Moffor F, Bukusi E. Cervical cancer prevention and treatment research in Africa: a systematic review from a public health perspective. BMC Womens Health. 2016;16:29 10.1186/s12905-016-0306-6 27259656 PMC4893293

[27] ICO/IARC Information Centre on HPV and Cancer: Chad, Human Papillomavirus and Related Cancers, Fact Sheet 2017. (Last accessed: July 2018). www.hpvcentre.net/statistics/reports/TCD_FS.pdf.

[28] Robin L, Mboumba Bouassa RS, Nodjikouambaye ZA, Charmant L, Matta M, Simon S et al Analytical performances of simultaneous detection of HIV-1, HIV-2 and hepatitis C- specific antibodies and hepatitis B surface antigen (HBsAg) by multiplex immunochromatographic rapid test with serum samples: A cross-sectional study. J Virol Methods. 2018;253:1–4. 10.1016/j.jviromet.2017.12.001 29208530

[29] Estrade C, Sahli R. Comparison of Seegene Anyplex II HPV28 with the PGMY-CHUV assay for human papillomavirus genotyping. J Clin Microbiol. 2014;52(2):607–12. 10.1128/JCM.02749-13 24478495 PMC3911335

[30] Bouvard V, Baan R, Straif K, Grosse Y, Secretan B, El Ghissassi F et al A review of human carcinogens--Part B: biological agents. Lancet Oncol. 2009 4;10(4):321–2. 19350698 10.1016/s1470-2045(09)70096-8

[31] Kwon MJ, Roh KH, Park H, Woo HY. Comparison of the Anyplex II HPV28 assay with the Hybrid Capture 2 assay for the detection of HPV infection. J Clin Virol. 2014;59(4):246–9. 10.1016/j.jcv.2014.01.015 24568964

[32] Marcuccilli F, Farchi F, Mirandola W, Ciccozzi M, Paba P, Bonanno E et al Performance evaluation of Anyplex II HPV28 detection kit in a routine diagnostic setting: comparison with the HPV Sign Genotyping Test. J Virol Methods. 2015;217:8–13. 10.1016/j.jviromet.2015.02.018 25724435

[33] Latsuzbaia A, Tapp J, Nguyen T, Fischer M, Arbyn M, Weyers S et al Analytical performance evaluation of Anyplex II HPV28 and Euroarray HPV for genotyping of cervical samples. Diagn Microbiol Infect Dis. 2016;85(3):318–322. 10.1016/j.diagmicrobio.2016.04.011 27156793

[34] Pasquier C, Sauné K, Raymond S, Boisneau J, Courtade M, Izopet J. Comparison of Cobas HPV and Anyplex II HPV28 assays for detecting and genotyping human papillomavirus. Diagn Microbiol Infect Dis. 2017;87(1):25–27. 10.1016/j.diagmicrobio.2016.08.022 28336133

[35] Szumilas M. Explaining odds ratios. J Can Acad Child Adolesc Psychiatry. 2010;19(3):227–9. 20842279 PMC2938757

[36] Petrelli A, Di Napoli A, Giorgi Rossi P, Rossi A, Luccini D, Di Marco I et al Prevalence of Primary HPV in Djibouti: Feasibility of Screening for Early Diagnosis of Squamous Intraepithelial Lesions. J Low Genit Tract Dis. 2016;20(4):321–6. 10.1097/LGT.0000000000000240 27467824

[37] Okunade KS, Nwogu CM, Oluwole AA, Anorlu RI. Prevalence and risk factors for genital high-risk human papillomavirus infection among women attending the out-patient clinics of a university teaching hospital in Lagos, Nigeria. Pan Afr Med J. 2017;28:227 10.11604/pamj.2017.28.227.13979 29629013 PMC5882206

[38] Sangwa-Lugoma G, Ramanakumar AV, Mahmud S, Liaras J, Kayembe PK, Tozin RR et al Prevalence and determinants of high-risk human papillomavirus infection in women from a sub-Saharan African community. Sex Transm Dis. 2011;38(4):308–15. 10.1097/OLQ.0b013e3181fc6ec0 21150817

[39] Kunckler M, Schumacher F, Kenfack B, Catarino R, Viviano M, Tincho E et al Cervical cancer screening in a low-resource setting: a pilot study on an HPV-based screen-and-treat approach. Cancer Med. 2017;6(7):1752–1761. 10.1002/cam4.1089 28580596 PMC5504339

[40] Catarino R, Vassilakos P, Tebeu PM, Schäfer S, Bongoe A, Petignat P. Risk factors associated with human papillomavirus prevalence and cervical neoplasia among Cameroonian women. Cancer Epidemiol. 2016;40:60–6. 10.1016/j.canep.2015.11.008 26625088

[41] Cubie HA, Morton D, Kawonga E, Mautanga M, Mwenitete I, Teakle N et al HPV prevalence in women attending cervical screening in rural Malawi using the cartridge-based Xpert HPV assay. J Clin Virol. 2017;87:1–4. 10.1016/j.jcv.2016.11.014 27984765

[42] Dartell M, Rasch V, Munk C, Kahesa C, Mwaiselage J, Iftner T et al Risk factors for high-risk human papillomavirus detection among HIV-negative and HIV-positive women from Tanzania. Sex Transm Dis. 2013;40(9):737–43. 10.1097/OLQ.0000000000000005 23949589

[43] Ngabo F, Franceschi S, Baussano I, Umulisa MC, Snijders PJ, Uyterlinde AM et al Human papillomavirus infection in Rwanda at the moment of implementation of a national HPV vaccination programme. BMC Infect Dis. 2016;16:225 10.1186/s12879-016-1539-6 27221238 PMC4877733

[44] Traore IMA, Zohoncon TM, Ndo O, Djigma FW, Obiri-Yeboah D, Compaore TR et al Oncogenic Human Papillomavirus Infection and Genotype Characterization among Women in Orodara, Western Burkina Faso. Pak J Biol Sci. 2016;19(7):306–311. 10.3923/pjbs.2016.306.311 29023032

[45] Traore IM, Zohoncon TM, Dembele A, Djigma FW, Obiri-Yeboah D, Traore G et al Molecular Characterization of High-Risk Human Papillomavirus in Women in Bobo-Dioulasso, Burkina Faso. Biomed Res Int. 2016;2016:7092583. 10.1155/2016/7092583 PMC4971308 27525275

[46] Catarino R, Vassilakos P, Jinoro J, Broquet C, Benski AC, Meyer-Hamme U et al Human papillomavirus prevalence and type-specific distribution of high- and low-risk genotypes among Malagasy women living in urban and rural areas. Cancer Epidemiol. 2016;42:159–66. 10.1016/j.canep.2016.04.013 27161432

[47] Ginindza TG, Dlamini X, Almonte M, Herrero R, Jolly PE, Tsoka-Gwegweni JM et al Prevalence of and Associated Risk Factors for High Risk Human Papillomavirus among Sexually Active Women, Swaziland. PLoS One. 2017;12(1):e0170189 10.1371/journal.pone.0170189 28114325 PMC5256897

[48] Mbulawa ZZ, Coetzee D, Williamson AL. Human papillomavirus prevalence in South African women and men according to age and human immunodeficiency virus status. BMC Infect Dis. 2015;15:459 10.1186/s12879-015-1181-8 26502723 PMC4624185

[49] Adler D, Laher F, Wallace M, Grzesik K, Jaspan H, Bekker LG et al High Rate of Multiple Concurrent Human Papillomavirus Infections among HIV-Uninfected South African Adolescents. J Immunol Tech Infect Dis. 2013;2(1):1000106 25333073 PMC4201846

[50] Watson-Jones D, Baisley K, Brown J, Kavishe B, Andreasen A, Changalucha J et al High prevalence and incidence of human papillomavirus in a cohort of healthy young African female subjects. Sex Transm Infect. 2013;89(5):358–65. 10.1136/sextrans-2012-050685 23486859 PMC3717757

[51] Ebrahim S, Mndende XK, Kharsany AB, Mbulawa ZZ, Naranbhai V, Frohlich J et al High Burden of Human Papillomavirus (HPV) Infection among Young Women in KwaZulu-Natal, South Africa. PLoS One. 2016 1 19;11(1):e0146603 10.1371/journal.pone.0146603 26785408 PMC4718633

[52] Schiffman M, Kjaer SK. Chapter 2: Natural history of anogenital human papillomavirus infection and neoplasia. J Natl Cancer Inst Monogr. 2003;(31):14–9. 10.1093/oxfordjournals.jncimonographs.a003476 12807940

[53] Akarolo-Anthony SN, Al-Mujtaba M, Famooto AO, Dareng EO, Olaniyan OB, Offiong R et al HIV associated high-risk HPV infection among Nigerian women. BMC Infect Dis. 2013;13:521 10.1186/1471-2334-13-521 24192311 PMC3826514

[54] Ezechi OC, Ostergren PO, Nwaokorie FO, Ujah IA, Odberg Pettersson K. The burden, distribution and risk factors for cervical oncogenic human papilloma virus infection in HIV positive Nigerian women. Virol J. 2014;11:5 10.1186/1743-422X-11-5 24433568 PMC3896716

[55] Adebamowo SN, Olawande O, Famooto A, Dareng EO, Offiong R, Adebamowo CA et al Persistent Low-Risk and High-Risk Human Papillomavirus Infections of the Uterine Cervix in HIV-Negative and HIV-Positive Women. Front Public Health. 2017;5:178 10.3389/fpubh.2017.00178 28785554 PMC5519520

[56] Chen Y, Bruning E, Rubino J, Eder SE. Role of female intimate hygiene in vulvovaginal health: Global hygiene practices and product usage. Womens Health (Lond). 2017 12;13(3):58–67. 28934912 10.1177/1745505717731011 PMC7789027

[57] Lewis FM, Bernstein KT, Aral SO. Vaginal Microbiome and Its Relationship to Behavior, Sexual Health, and Sexually Transmitted Diseases. Obstet Gynecol. 2017 4;129(4):643–654. 10.1097/AOG.0000000000001932 28277350 PMC6743080

[58] Meader N, King K, Moe-Byrne T, Wright K, Graham H, Petticrew M et al. A systematic review on the clustering and co-occurrence of multiple risk behaviours. BMC Public Health. 2016 7 29;16:657 10.1186/s12889-016-3373-6 27473458 PMC4966774

[59] World Health Organization (WHO). Human papillomavirus vaccines: WHO position paper, October 2014. (Last accessed: December 2018). http://www.who.int/wer/2014/wer8943.pdf?ua=1.

[60] UNAIDS. UNAIDS data 2017. July 2017. (Last accessed: December 2018). http://www.unaids.org/en/resources/documents/2017/2017_data_book.

[61] Bessimbaye N, Moussa AM, Mbanga D, Tidjani A, Mahamat SO, Ngawara MN et al [Seroprevalence of HBs Ag and of anti-HCV antibodies among HIV infected people in N’Djamena, Chad]. Bull Soc Pathol Exot. 2014;107(5):327–31. 10.1007/s13149-014-0386-1 25158842

[62] Teunis N. Same-Sex Sexuality in Africa: A Case Study from Senegal AIDS Behav. 2001, 5 (2):173–182. (Last accessed: December 2018). Available at: 10.1023/A:1011335129358.

